# Divergent Modulation of Nociception by Glutamatergic and GABAergic Neuronal Subpopulations in the Periaqueductal Gray

**DOI:** 10.1523/ENEURO.0129-16.2017

**Published:** 2017-03-29

**Authors:** Vijay K. Samineni, Jose G. Grajales-Reyes, Bryan A. Copits, Daniel E. O’Brien, Sarah L. Trigg, Adrian M. Gomez, Michael R. Bruchas, Robert W. Gereau

**Affiliations:** 1 Washington University Pain Center and Department of Anesthesiology, Washington University School of Medicine, St. Louis, MO 63110; 2 Medical Scientist Training Program, Washington University School of Medicine, St. Louis, MO 63110; 3 Division of Biomedical and Biological Sciences Graduate Program in Neuroscience, Washington University School of Medicine, St. Louis, MO 63110; 4 Department of Neuroscience, Washington University School of Medicine, St. Louis, MO 63110; 5 Washington University School of Medicine, St. Louis, MO 63110

**Keywords:** chemogenetics, Descending modulation, DREADDs, PAG, pain, RVM

## Abstract

The ventrolateral periaqueductal gray (vlPAG) constitutes a major descending pain modulatory system and is a crucial site for opioid-induced analgesia. A number of previous studies have demonstrated that glutamate and GABA play critical opposing roles in nociceptive processing in the vlPAG. It has been suggested that glutamatergic neurotransmission exerts antinociceptive effects, whereas GABAergic neurotransmission exert pronociceptive effects on pain transmission, through descending pathways. The inability to exclusively manipulate subpopulations of neurons in the PAG has prevented direct testing of this hypothesis. Here, we demonstrate the different contributions of genetically defined glutamatergic and GABAergic vlPAG neurons in nociceptive processing by employing cell type-specific chemogenetic approaches in mice. Global chemogenetic manipulation of vlPAG neuronal activity suggests that vlPAG neural circuits exert tonic suppression of nociception, consistent with previous pharmacological and electrophysiological studies. However, selective modulation of GABAergic or glutamatergic neurons demonstrates an inverse regulation of nociceptive behaviors by these cell populations. Selective chemogenetic activation of glutamatergic neurons, or inhibition of GABAergic neurons, in vlPAG suppresses nociception. In contrast, inhibition of glutamatergic neurons, or activation of GABAergic neurons, in vlPAG facilitates nociception. Our findings provide direct experimental support for a model in which excitatory and inhibitory neurons in the PAG bidirectionally modulate nociception.

## Significance Statement

The periaqueductal gray (PAG) is a midbrain region critical for the modulation of pain. However, the roles played by the distinct cell types within the PAG in nociceptive processing are poorly understood. This work addresses the divergent roles of glutamatergic and GABAergic PAG neuronal subpopulations in nociceptive processing. We demonstrate that activation of glutamatergic neurons or inhibition of GABAergic neurons suppresses nociception. However, inhibition of glutamatergic neuronal activity or activation of GABAergic neuronal activity potentiates nociception. This report identifies distinct roles for these neuronal populations in modulating nociceptive processing.

## Introduction

The periaqueductal gray (PAG), an evolutionarily conserved neurosubstrate in the midbrain, regulates a wide of complex behaviors, including pain (Basbaum and Fields, 1978; Graeff et al., 1993; Behbehani, 1995; Bandler and Keay, 1996; Holstege, 2014; Tovote et al., 2016; Watson et al., 2016). The ventrolateral PAG (vlPAG) is a major site of endogenous opioid-induced pain suppression, and electrical stimulation of the vlPAG produces profound analgesia (Reynolds, 1969; Liebeskind et al., 1973; Mayer and Liebeskind, 1974; Hosobuchi et al., 1977; Baskin et al., 1986; Fields, 2004). The robust modulatory role of the vlPAG on spinal nociceptive processing is mediated by descending projections from the vlPAG via the rostral ventromedial medulla (RVM; Liebeskind et al., 1973; Basbaum and Fields, 1979; Bennett and Mayer, 1979; Hayes et al., 1979; Beitz et al., 1983; Duggan and Morton, 1983; Watkins et al., 1983; Morton et al., 1984; Moreau and Fields, 1986; Morgan et al., 1989; Urban and Smith, 1994; Pertovaara et al., 1996; Waters and Lumb, 1997; Antal and Odeh, 1998; Budai and Fields, 1998; Odeh and Antal, 2001; Maione et al., 2006; Waters and Lumb, 2008).

Previous studies have suggested that the vlPAG has a bidirectional role in the modulation of nociception. Nonspecific activation of vlPAG neurons produces analgesia, whereas inhibition of vlPAG produces hyperalgesia to noxious stimulation (Reynolds, 1969; Liebeskind et al., 1973; Moreau and Fields, 1986; Depaulis et al., 1987; Siegfried and de Souza, 1989). It is tempting to speculate that output from the vlPAG has a purely analgesic action. However, the vlPAG comprises diverse subpopulations of neurons with distinct neurochemical properties that regulate excitatory and inhibitory neurotransmission (Behbehani and Fields, 1979; Moss and Basbaum, 1983; Moss et al., 1983; Moreau and Fields, 1986; Behbehani et al., 1990; Behbehani, 1995; Vaughan et al., 1997; Hahm et al., 2011; Ho et al., 2013). Microinjection of glutamate receptor agonists or GABA antagonists into the vlPAG leads to global activation of neurons and produces antinociceptive effects to noxious stimuli (Moreau and Fields, 1986; Ness and Gebhart, 1987; Carstens et al., 1988; Jacquet, 1988; Jones and Gebhart, 1988; Jensen and Yaksh, 1989; Sandkühler et al., 1989; Carstens et al., 1990; Budai and Fields, 1998; Morgan et al., 2003). In contrast, microinjecting glutamatergic antagonists or GABA agonists, presumably leading to global suppression of neural activity in the vlPAG, produces hyperalgesia (Moreau and Fields, 1986; Depaulis et al., 1987; Siegfried and de Souza, 1989; Behbehani et al., 1990). These studies suggest that in the context of a noxious stimulus, GABAergic neurotransmission in the vlPAG is pronociceptive, although the source of the GABAergic inputs to the vlPAG cannot be determined (Reichling and Basbaum, 1990a, 1990b). Collectively, a large number of prior studies suggest that glutamatergic and GABAergic neurons within the vlPAG play critical and complex roles in processing nociception (Behbehani and Fields, 1979; Moreau and Fields, 1986; Millan et al., 1987; Sandkühler et al., 1989; Siegfried and de Souza, 1989; Vaughan et al., 1997). Based on these studies, the vlPAG GABA disinhibition hypothesis has been proposed (Basbaum and Fields, 1978; Fields, 2004; Lau and Vaughan, 2014). In this hypothesis, GABAergic interneurons exert tonic inhibition over vlPAG glutamatergic neurons, which are thought to be output neurons that project to the RVM to facilitate the descending inhibition of nociception (Vaughan et al., 1997; Budai and Fields, 1998; Wang and Wessendorf, 2002; Maione et al., 2006; Starowicz et al., 2007; Heinricher et al., 2009; Park et al., 2010; Tovote et al., 2016). Despite the wealth of evidence supporting this model, the distinct roles of GABAergic and glutamatergic neuronal populations in descending vlPAG pain modulation have not been directly investigated. In this study, we use cell type-specific chemogenetic manipulations of neuronal activity in the vlPAG to test the hypothesis that GABAergic neurons are pronociceptive and glutamatergic neurons are antinociceptive.

## Materials and Methods

### Animals

All experiments were conducted in accordance with the National Institutes of Health guidelines and with approval from the Animal Care and Use Committee of Washington University School of Medicine. Male, 8- to 12-week-old, heterozygous *Slc32a1^tm2L^°^wl^* (Vgat-ires-Cre, selectively targets Vgat^+^ GABAergic inhibitory neurons), *Slc17a6^tm2L^°^wl^* (Vglut2-ires-Cre, selectively targets Vglut2^+^ glutamatergic excitatory neurons, and C57BL\6J mice were used (Vong et al., 2011). Mice were purchased from Jackson Laboratories (C57BL\6J, Vgat Cre, stock number 016962 and Vglut2 Cre stock number 016963) and colonies were established in our facilities. Experimenters were blind to treatment and genotype.

### Viral constructs and surgery

Adeno-associated viruses (AAV8) were used to achieve Cre-independent chemogenetic vector expression: hM3Dq-mCherry (rAAV8/hSyn-hM3Dq-mCherry; 3.2 × 10^12^ particles/ml), hM4Di-mCherry (rAAV8/hSyn-hM4Di-mCherry; 2 × 10^12^ particles/ml), and control eGFP (rAAV8/hSyn-eGFP; 8 × 10^12^ particles/ml). Adeno-associated viruses (AAV5) were used to achieve Cre-dependent vector expression: hM3Dq-mCherry (rAAV5/hSyn-DIO-hm3Dq-mCherry; 6 × 10^12^ particles/ml), hM4Di-mCherry (rAAV5/hSyn-DIO-hm4Di-mCherry; 6 × 10^12^ particles/ml), and control eGFP (rAAV5/hSyn-DIO-eGFP; 3.4 × 10^12^ particles/ml). All viral vectors were acquired from the University of North Carolina Vector Core Facility. Before surgery, mice were anesthetized with isoflurane and secured in a stereotactic frame (David Kopf Instruments). A small midline dorsal incision was performed to expose the skull and bilateral viral injections were performed using the following coordinates: vlPAG, −4.8–4.9 mm from bregma, ±0.3–0.4 mm lateral from midline, and 2.7–2.9 mm ventral to skull. Injections of 150 nL of the desired viral vectors into the vlPAG were performed at a rate of 100 nl per 60 s.

### Chemogenetic manipulation

Three weeks after viral injections, mice were injected intraperitoneally with clozapine N-oxide (CNO, BML-NS105 from Enzo life sciences) 60 min before beginning behavioral assessment, and data were collected between the second and third hour after injection. All baselines for thermal and mechanical sensitivity were recorded two weeks after the viral injections and one week before the CNO administration. The doses of CNO were chosen based on preliminary pilot experiments designed to determine the minimal dose needed to activate designer receptor exclusively activated by designer drugs (DREADD) receptors in the vlPAG of C57BL\6J mice, Vgat-ires-Cre and Vglut2-ires-Cre mice. We assessed the response of these different mouse lines to various doses of CNO to identify the minimal doses required to activate DREADDs (data not shown). In non-Cre-dependent studies, we administered 1 mg/kg CNO for both hM3Dq activation and hM4Di inhibition. In Vgat Cre mice, we administered 3 mg/kg CNO for both hM3Dq activation and hM4Di inhibition. In Vglut2 Cre mice, we administered 2 mg/kg CNO for both hM3Dq activation and hM4Di inhibition.

### Pain behavior assessment

To evaluate nociception, mechanical withdrawal thresholds and thermal withdrawal latencies were assayed. Mice were tested for baseline responses to mechanical and thermal stimuli, as previously described (O'Brien et al., 2013). For the assessment of mechanical withdrawal threshold, von Frey filaments (North Coast Medical) were applied bilaterally to the hind paws of the mice using the up-down method. Two to three trials were performed on each hind paw for each mouse. The average 50% withdrawal threshold was calculated for each paw individually and then averaged to obtain a threshold value for each mouse. The Hargreaves test was performed to evaluate heat sensitivity thresholds, measuring latency of withdrawal to a radiant heat source (IITC Life Science, Model 390). We applied the radiant heat source bilaterally to the hind paw and measured the latency to evoke a withdrawal. Three to five replicates were acquired per hind paw per mouse, and values for both paws were averaged.

### Electrophysiology

To determine the functional effects of chemogenetic manipulations in vlPAG neurons, we performed targeted whole-cell patch-clamp recordings in acute coronal slices from both Vgat- and Vglut2-Cre mice expressing either hM3Dq or hM4Di receptors. Mice used for electrophysiology and behavioral studies were between 8 and 12 weeks of age. Three weeks after viral infection of vlPAG neurons, coronal slices containing the vlPAG were prepared as previously described (Siuda et al., 2015). GABAergic and glutamatergic neurons in the vlPAG were visualized through a 40× objective using IR-differential interference contrast (DIC) microscopy on an Olympus BX51 microscope, and mCherry+ cells were identified using epifluorescent illumination with a green LED (530 nm; Thorlabs), coupled to the back fluorescent port of the microscope. Whole-cell recordings of vlPAG GABAergic and glutamatergic neurons expressing hM3Dq-mCherry and hM4Di-mCherry were performed using a Heka EPC 10 amplifier (Heka) with Patchmaster software (Heka). Following stable 5-min whole-cell recordings (baseline), the direct effects of either hM3Dq or hM4Di receptor expression on cellular excitability was isolated by blocking AMPA/KARs (10 µM NBQX, Abcam), NMDARs (50 µM D-APV, Abcam), GABA_A_Rs (100 µM picrotoxin, Abcam), and GABA_B_Rs (50 µM baclofen, Abcam). aCSF solution containing 10 µM CNO added to the antagonist cocktail above was bath applied to the brain slice.

### Immunohistochemistry

To perform histologic confirmation of virus expression and injection sites, C57, Vgat Cre, and Vglut2 Cre mice expressing hM3Dq-mCherry, hM4Di-mCherry, and EGFP virus were deeply anesthetized with ketamine/xylazine cocktail at the end of every experiment and then perfused with 20 ml of PBS and 20 ml of a 4% paraformaldehyde PBS solution (PFA). Brains were removed, postfixed in 4% PFA overnight at 4°, and then immersed in 30% sucrose for cryoprotection. Using a cryostat, 30-μm tissue sections were collected and stored in PBS, containing 0.4% sodium azide, at 4°. After washing the sections in PBS 1×, we incubated the tissues in blocking solution containing 5% normal goat serum and 0.2% Triton X-100 PBS solution for 1 h at room temperature. Primary antibodies against mCherry (Mouse, Clontech 632392; 1:500) and GFP (rabbit polyclonal, Life Technologies A11122, 1:500) were diluted in blocking solution and incubated overnight at 4°C. After three 10-min washes, tissues were incubated for 1 h at room temperature with secondary antibodies [Life Technologies: Alexa Fluor 488 donkey anti rabbit IgG (1:300); Alexa Fluor 488 goat anti rabbit (1:300); Alexa Fluor 555 goat anti mouse (1:300); and Neurotrace (435/455nm, 1:500)]. Sections were mounted with Vectashield (H-1400) hard mounting media and imaged on a Nikon Eclipse 80i epifluorescence microscope.

### Fluorescence in situ hybridization (FISH)

Following rapid decapitation of mice, brains were flash frozen in −50°C 2-methylbutane and stored at −80°C for further processing. Coronal sections containing the PAG, corresponding to the injection coordinates used in the behavioral experiments, were cut at 20 μm at −20°C and thaw-mounted onto Super Frost Plus slides (Fisher). Slides were stored at -80°C until further processing. FISH was performed according to the RNAScope 2.0 Fluorescent Multiple Kit User Manual for Fresh Frozen Tissue (Advanced Cell Diagnostics) as described previously (Wang et al., 2012). Slides containing PAG coronal brain sections were fixed in 4% PFA, dehydrated, and pretreated with protease IV solution for 30 min. Sections were then incubated with target probes for mouse Vglut2 (*slc17a6*, accession number NM_080853.3, probe region 1986-2998), Vgat (*slc32a1*, accession number NM_009508.2, probe region 894-2037), and Cre (accession number KC845567.1, probe region 1058-2032). All target probes consisted of 20 double Z oligonucleotides and were obtained from Advanced Cell Diagnostics. Following probe hybridization, sections underwent a series of probe signal amplification steps (AMP1-4) followed by incubation of fluorescent probes (Alexa Fluor 488, Atto 550, Atto 647), designed to target the specified channel associated with the probes. Slides were counterstained with 4',6-diamidino-2-phenylindole (DAPI; RNAScope), and coverslips were mounted with Vectashield Hard Set mounting medium (Vector Laboratories). Images were obtained on a Leica TCS SPE confocal microscope (Leica), and Application Suite Advanced Fluorescence (LAS AF) software was used for analyses.

### Statistics

Throughout the study, researchers were blinded to all experimental conditions. At least two to three replicate measurements were performed and averaged in all behavioral assays. The studies were designed to compare behavioral readouts following CNO to baseline values before CNO administration. This was done using paired *t* tests to account for interindividual variability among mice across different cohorts. The control eGFP group was included throughout our study to determine whether CNO administration or DREADD expression had off-target effects in the behaviors that we tested. All datasets were evaluated for normality using the D’Agostino and Pearson omnibus normality test. A parametric test was used only when normality was confirmed. If normality could not be confirmed, the nonparametric Wilcoxon matched pairs test was used.

## Results

### Global chemogenetic manipulation of vlPAG activity suggests bidirectional modulation of nociceptive behaviors

**Figure 1.**
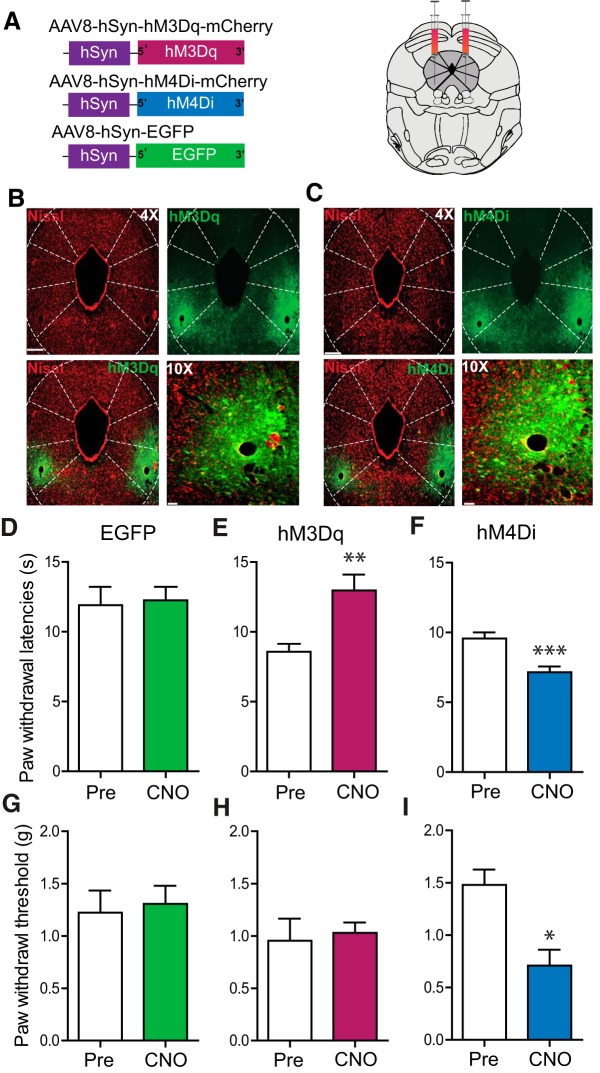
Global chemogenetic manipulation of vlPAG activity suggests parallel bidirectional modulation of nociceptive behaviors. ***A***, Constructs used in viral targeting of AAV8 hM3Dq–mCherry, AAV8 hM4Di–mCherry and AAV8–EGFP via bilateral injections into the vlPAG. ***B***, ***C***, Representative images of coronal sections containing vlPAG demonstrating restricted viral expression following microinjection of the AAV8 hM3Dq (***B***) and hM4Di (***C***) into the vlPAG. ***D***, ***G***, Relative to pretreatment baseline values, CNO (1 mg/kg, i.p.) did not have any significant effects on PWLs in mice expressing the control EGFP construct. ***E***, ***H***, CNO (1 mg/kg, i.p.) administration in hM3Dq-injected mice resulted in a significant increase in PWLs but not in PWTs. ***F***, ***I***, CNO (1 mg/kg, i.p.) administration in hM4Di-injected mice resulted in a significant decrease in PWLs and PWTs. ∗*p* < 0.05, ∗∗*p* < 0.005, ∗∗∗*p* < 0.0001. Scale bars, 300 and 35 μm, 4× and 10×, respectively.

We used a chemogenetic approach to investigate whether selectively manipulating activity of resident neurons in the vlPAG can modulate different nociceptive modalities. DREADDs exploit selective expression of mutated muscarinic receptors that are responsive to an exogenously administered, normally inert ligand, CNO (Rogan and Roth, 2011). Adeno-associated virus type 8 (AAV8), carrying neuron-specific stimulatory (hM3Dq) or inhibitory (hM4Di) DREADD fused with mCherry, was microinjected bilaterally into the vlPAG (Fig. 1*A*). Robust expression of DREADDs, restricted to the vlPAG, was observed three weeks after AAV8/hSyn-hM3Dq-mCherry (Fig. 1*B*) or AAV8/hSyn-hM4Di-mCherry injection (Fig. 1*C*). In mice expressing the stimulatory DREADD (hM3Dq) in vlPAG neurons, CNO (1 mg/kg, i.p.) injection resulted in a significant increase in paw withdrawal latencies (PWLs) to thermal stimulation compared with baseline PWLs before CNO administration but did not alter paw withdrawal thresholds (PWTs) to mechanical stimuli compared with baseline PWTs before CNO administration (Fig. 1*E*; *t*_(10)_ = 3.674, **p = 0.0043, *n* = 11; Fig. 1*H*; *t*_(10)_ = 0.3489, *p* = 0.73, *n* = 11). On the other hand, in mice expressing the inhibitory DREADD (hM4Di) in vlPAG neurons, CNO (1 mg/kg, i.p.) injection resulted in a significant decrease in PWLs and PWTs compared with baseline (Fig. 1*F*; *t*_(11)_ = 6.693, ***p < 0.0001, *n* = 12; Fig. 1*I*; *p < 0.05, *n* = 11), indicating development of thermal and mechanical hypersensitivity. To confirm that CNO administration did not have any off-target effects on PWLs and PWTs, control mice expressing eGFP were administered CNO (1 mg/kg), which had no effect on PWLs or PWTs when compared with baseline (Fig. 1*D*; *t*_(11)_ = 0.2572, *p* = 0.80, *n* = 12; Fig. 1*G*, *t*_(8)_ = 0.2945, *p* = 0.77, *n* = 9). Taken together, these findings demonstrate that globally activating vlPAG neurons attenuates nociception, while inhibiting them potentiates nociception, consistent with prior studies using pharmacologic activation or inhibition of the vlPAG neurons (Moreau and Fields, 1986; Carstens et al., 1988; Jones and Gebhart, 1988; Jensen and Yaksh, 1989; Siegfried and de Souza, 1989; Behbehani et al., 1990; Carstens et al., 1990; Vaughan et al., 1997; Budai and Fields, 1998; Morgan et al., 2003).

### The role of vlPAG GABAergic and glutamatergic neuronal populations in nociceptive processing

**Figure 2.**
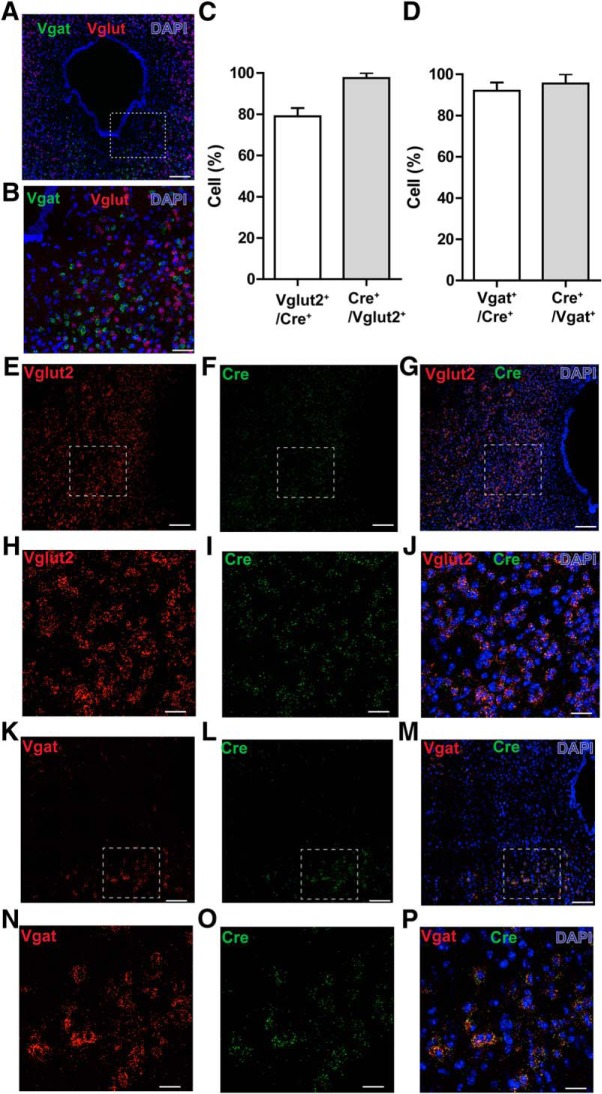
RNA-FISH demonstrates segregation of vlPAG GABAergic and glutamatergic neurons and specificity of Cre in targeting *Vgat+* neurons in the Vgat-IRES-Cre mice or *Vglut2+* neurons in the Vglut2-IRES-Cre mice. ***A***, Double RNA-FISH for *Vgat* (green) and *Vglut2* (red) shows that GABAergic and glutamatergic neurons in the PAG are nonoverlapping populations. Scale bar, 200 μm. Counterstaining (blue) is DAPI. ***B***, High-magnification image showing no colocalization of GABAergic and glutamatergic neurons in the PAG. Scale bar, 60 μm. ***C***, 79 ± 4.1% of cells positive for *Vglut2* transcripts in the vlPAG colabel with Vglut2 Cre-expressing neurons, and 97.5 ± 2.5% of Vglut2 Cre-expressing neurons in the vlPAG colabel with *Vglut2^+^* transcripts (*N* = 2 mice). ***D***, 92 ± 4.5% of cells positive for *Vgat* transcripts in the vlPAG colabel with Vgat Cre-expressing neurons, and 95.5 ± 4.2% of Vgat Cre-expressing neurons in the vlPAG colabel with *Vgat^+^* transcripts (*N* = 2 mice). ***E–G***, Double RNA-FISH for *Vglut2* (red) and Cre (green) shows extensive colocalization of *Vglut2* ^+^ transcripts with Cre-expressing neurons in the vlPAG obtained from Vglut2 Cre mice. Scale bar, 60 μm. ***H–J***, High-magnification image shows extensive colocalization of *Vglut2^+^* transcripts with Cre-expressing neurons in the vlPAG. Scale bar, 15 μm. ***K***, ***M***, Double RNA-FISH for *Vgat* (red) and Cre (green) shows extensive colocalization of *Vgat^+^* transcripts with Cre-expressing neurons in the vlPAG obtained from Vgat Cre mice. Scale bar, 60 μm. ***N–P***, High-magnification image shows extensive colocalization of *Vgat^+^* transcripts with Cre-expressing neurons in the vlPAG. Scale bar, 15 μm.

The vlPAG is comprised of both inhibitory GABAergic and excitatory glutamatergic neurons, and we hypothesized that these neuronal populations differentially regulate nociceptive processing. Double-label RNA-FISH in the vlPAG of c57-mice revealed that GABAergic neurons (*Vgat* transcripts) and glutamatergic neurons (*Vglut2* transcripts) show no overlap in expression in the PAG and, thus, are distinct populations (Fig. 2*A*,*B*). To selectively test the functional contributions of vlPAG GABAergic and glutamatergic neurons in modulating nociceptive behaviors, we used Vgat-ires-Cre and Vglut2-IRES-Cre mice to target and manipulate the activity of GABAergic and glutamatergic neurons, respectively. To determine the specificity of Cre in targeting *Vgat^+^* neurons in Vgat-IRES-Cre mice or in targeting *Vglut2^+^* neurons in the Vglut2-IRES-Cre mice, we performed RNA-FISH using probes for Vgat, Vglut, and Cre in vlPAG slices obtained from Vgat Cre and Vglut2 Cre mice. We observed 79 ± 4.1% of *Vglut2^+^* transcripts in the vlPAG colabel with Vglut2 Cre-expressing neurons, and 97.5 ± 2.5% Vglut2 Cre-expressing neurons in the vlPAG colabel with *Vglut2^+^* transcripts (Fig. 2*C*,*E–J*). We also observed 92 ± 4.5% of *Vgat^+^* transcripts in the vlPAG colabel with Vgat Cre-expressing neurons, and 95.5 ± 4.2% Vgat Cre-expressing neurons in the vlPAG colabel with *Vgat^+^* transcripts (Fig. 2*D*,*K*–*P*). Our double-label RNA-FISH studies revealed that Vgat and Vglut2 Cre mice faithfully label vlPAG GABAergic and Vglut2^+^ glutamatergic neuronal populations, as described previously for other brain regions (Vong et al., 2011).

**Figure 3.**
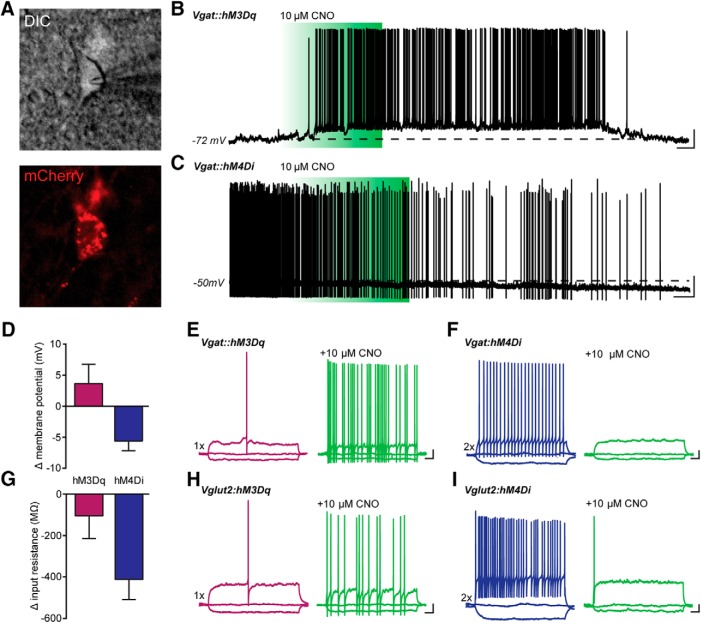
Functional characterization of G_q_- and G_i_-DREADDs in vlPAG neurons of Vgat-Cre and Vglut2-Cre mice. ***A***, Infrared DIC image of vlPAG Vgat^+^ neuron expressing hM4Di-mCherry. Images were acquired following CNO stimulation. ***B***, Whole-cell current-clamp recording from an hM3Dq-expressing PAG neuron. Brief bath application of 10 µM CNO caused a transient depolarization and robust action potential firing in Vgat^+^ and Vglut^+^ neurons. ***C***, Voltage trace showing that bath perfusion with 10 µM CNO caused prolonged membrane hyperpolarization and silencing of both Vgat^+^ and Vglut^+^ vlPAG neurons. Dashed lines in ***B*** and ***C*** represent the membrane potential of the cells before application of CNO. ***D***, ***G***, Quantification of the CNO effects on membrane potential and input resistance in grouped Vgat^+^ and Vglut2^+^ neurons (*N* = 8 for Vgat^+^ and Vglut2^+^ neurons). ***E–I***, Voltage traces showing responses to a hyperpolarizing current of -20 pA and a depolarizing current injection of either 1× rheobase (purple traces) or 2× rheobase (blue traces) in both Vgat^+^ (***E***, ***H***) and Vglut2^+^ (***F***, ***I***) neurons. In hM3Dq-expressing neurons, bath application of CNO elicited increased action potential firing in response to the same stimulus (***E***, ***H***, green traces). In hM4Di^+^ neurons, CNO perfusion decreased neuronal excitability to supratheshold stimuli. ***B***, ***C***, Scale bars, 20 mv and 10 s; ***E–I***, Scale bars, 10 mv and 100 ms. All values are mean ± SEM.

To selectively test the functional contributions of vlPAG GABAergic and glutamatergic neurons in modulating nociceptive behaviors, we used Cre-dependent DREADD expression in Vgat-ires-Cre and Vglut2-ires-Cre mice to target GABAergic and glutamatergic neurons, respectively. Virus carrying Cre-dependent stimulatory (hM3Dq) or inhibitory (hM4Di) DREADDs fused with mCherry were injected into the vlPAG of Vgat-Cre mice or Vglut2-Cre mice. Three weeks after DREADD injection, we prepared acute coronal slices of the vlPAG from Vgat-Cre and Vglut2-Cre mice and targeted mCherry^+^ neurons in vlPAG for whole-cell recordings (Fig. 3*A*). hM3Dq-expressing vlPAG neurons were held at hyperpolarized membrane potentials, and a brief bath application of 10 μM CNO caused a transient depolarization and robust action potential firing in both Vgat and Vglut2 neurons (Fig. 3*B*). To test the effects of G_i_-coupled inhibition with hM4Di, we monitored neuronal activity while holding cells with a depolarizing current injection, which elicited persistent action potential firing in both Vgat and Vglut2 neurons. Bath perfusion with 10 μM CNO resulted in prolonged membrane hyperpolarization and decreased firing of both cell types (Fig. 3*C*). Quantification of hM3Dq-expressing neurons showed that CNO depolarized neurons by an average of 3.6 mV and caused a small decrease in the input resistance (Fig. 3*D*,*G*). In contrast, activation of hM4Di hyperpolarized neurons by an average of 5.6 mV, and substantially reduced input resistance, consistent with CNO-induced G_i_-coupling to inwardly rectifying K^+^ channels (Sternson and Roth, 2014; Urban and Roth, 2015; Fig. 3*D*,*G*).

We also investigated how CNO modulates membrane excitability in response to depolarizing step current injections. In hM3Dq-expressing neurons, we observed a large increase in the number of action potentials elicited during a 1× rheobase current following CNO stimulation in slices from both Vgat- and Vglut2-Cre mice (Fig. 3*E*,*H*). Suprathreshold current injections of 2× rheobase elicited sustained high-frequency action potential firing in both neuronal subtypes (Fig. 3*F*,*I*, blue traces), and CNO application dramatically reduced membrane excitability to an identical suprathreshold stimulus in mice injected with hM4Di constructs (Fig. 3*F*,*I*, green traces). This confirmed that we are able to bi-directionally modulate GABAergic and glutamatergic neuron excitability in the vlPAG using hM3Dq and hM4Di DREADDs, and led us to explore the contributions of these neuronal populations in nociceptive processing.

**Figure 4.**
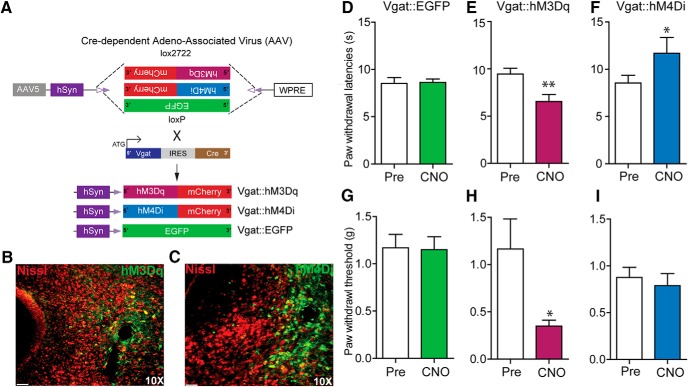
Chemogenetic manipulation of vlPAG GABAergic neurons bidirectionally modulates nociceptive behaviors. ***A***, Illustration showing viral targeting strategy of AAV5-hSyn-DIO-hM3Dq–mCherry, AAV5-hSyn-DIO-hM4Di–mCherry, and AAV5-hSyn-DIO-EGFP bilaterally injected into the vlPAG of Vgat Cre mice. ***B***, ***C***, Representative images of coronal sections containing vlPAG showing restricted viral expression following microinjection of AAV5-hSyn-DIO-hM3Dq (***B***) or AAV5-hSyn-DIO-hM4Di (***C***) into the vlPAG of Vgat Cre mice. ***E***, ***H***, CNO (3 mg/kg, i.p.) administration resulted in a significant decrease in PWLs and PWTs in Vgat::hM3Dq mice. ***F***, ***I***, CNO administration resulted in a significant increase in PWLs but not in PWTs in Vgat::hM4Di mice. ***D***, ***G***, CNO had no significant effect on PWLs or PWTs in Vgat Cre mice expressing the control EGFP construct compared with baseline PWLs and PWTs before CNO administration. All values are mean ± SEM. Student’s *t* test; ∗*p* < 0.05, ∗∗*p* < 0.005. Scale bars, 25 μm.

To assess how vlPAG GABAergic neurons contribute to nociceptive processing, we introduced Cre-dependent viral constructs containing either hM3Dq or hM4Di fused to mCherry or control virus lacking the DREADDs (hSyn-DIO-eGFP) into the vlPAG of Vgat-IRES-Cre mice (Fig. 4*A*). Three weeks after viral infection of vlPAG neurons, restricted expression of the DREADD vectors was observed in neurons within the vlPAG (Fig. 4*B*,*C*). CNO-dependent (3 mg/kg, i.p.) activation of vlPAG GABAergic neurons via hM3Dq resulted in a significant decrease in PWLs to a noxious thermal stimulus (Fig. 4*E*; *t*_(8)_ = 4.403, *p* = 0.0023, *n* = 9) and PWTs to a mechanical stimulus (Fig. 4*H*; *p* = 0.0469, *n* = 7) compared with baseline. In contrast, CNO-induced (3 mg/kg, i.p.) inhibition of vlPAG GABAergic neurons expressing hM4Di resulted in a significant increase in PWLs to noxious thermal stimulation compared with baseline (Fig. 4*F*, *t*_(13)_ =2.459, *p* = 0.0287, *n* = 14) but did not have a significant effect on PWTs to mechanical stimuli compared with baseline (Fig. 4*I*; *t*_(11)_ = 0.5885, *p* = 0.5681, *n* = 12). CNO (3 mg/kg, i.p.) administration did not affect PWLs or PWTs in control mice expressing DIO-EGFP when compared with baseline (Fig. 4*D*; *t*_(9)_ = 0.1837, *p* = 0.8584, *n* = 10) and (Fig. 4*G*; *t*_(8)_ = 0.2055, *p* = 0.8423, *n* = 9). Taken together, these results demonstrate that activation of GABAergic vlPAG neurons results in hypersensitivity to mechanical and noxious thermal stimuli, while inhibiting the activity of GABAergic vlPAG neurons decreases sensitivity to noxious heat only.

**Figure 5.**
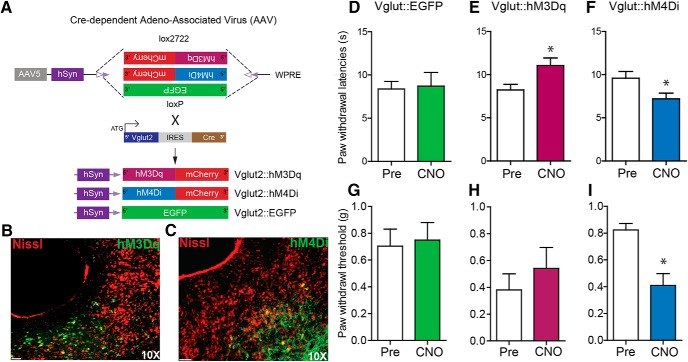
Chemogenetic modulation of vlPAG glutamatergic neurons bidirectionally modulates nociceptive behaviors. ***A***, Illustration showing the strategy for viral targeting of AAV5-hSyn-DIO-hM3Dq–mCherry, AAV5-hSyn-DIO-hM4Di–mCherry, and AAV5-hSyn-DIO-EGFP bilaterally injected into the vlPAG of Vglut2 Cre mice. ***B***, ***C***, Representative images of a coronal sections containing vlPAG showing restricted viral expression following microinjection of the AAV5-hSyn-DIO-hM3Dq–mCherry and AAV5-hSyn-DIO-hM4Di–mCherry into the vlPAG of Vglut2 Cre mice. ***E***, ***H***, CNO (2 mg/kg, i.p.) administration resulted in a significant increase in PWLs but not on PWTs in Vglut2::hM3Dq mice. ***F***, ***I***, CNO (2 mg/kg, i.p.) administration resulted in a significant decrease in PWLs and PWTs in Vglut2::hM4Di mice. ***D***, ***G***, CNO had no significant effect on PWLs or PWTs in Vglut2 Cre mice expressing the control EGFP construct compared with baseline PWLs and PWTs before CNO administration. All values are mean ± SEM, Student’s *t* test; ∗*p* < 0.05. Scale bars, 25 μm.

To directly examine the role of vlPAG glutamatergic neurons in nociceptive processing, Cre-dependent viral constructs carrying hM3Dq-mCherry, hM4Di-mCherry, or a control virus (hSyn-DIO-eGFP) were injected bilaterally into the vlPAG of Vglut2-ires-Cre mice (Fig. 5*A*). Three weeks after injection, we observed robust DREADD expression restricted to the vlPAG (Fig. 5*B*,*C*). Chemogenetic activation of vlPAG Vglut2 neurons expressing hM3Dq with CNO (2 mg/kg, i.p.) significantly increased PWLs to thermal stimuli compared with baselines before CNO administration (Fig. 5*E*; *t*_(11)_ =2.375, *p* = 0.0368, *n* = 12) but did not significantly alter PWTs to mechanical stimuli compared with baseline (Fig. 5*H*; *t*_(8)_ = 0.8779, *p* = 0.405, *n* = 9). To further examine the functional role of intrinsic activity of vlPAG Vglut2 neurons, we administered CNO (2 mg/kg, i.p.) to inhibit vlPAG Vglut2 neurons expressing hM4Di. This resulted in a significant decrease in PWLs to a noxious heat stimulus compared with baseline (Fig. 5*F*; *n* = 6, paired *t* test, *p* = 0.0313) and a decrease in PWTs to a mechanical stimulus compared with baseline (Fig. 5*I*; *n* = 6, paired *t* test, *p* = 0.0211). Control virus-injected mice showed no alterations in PWTs or PWLs on CNO (2 mg/kg, i.p.) administration compared with baseline (Fig. 5*D*; *t*_(9)_ = 0.2897 *p* = 0.7786, *n* = 10; Fig. 5*G*, *p* = 1, *n* = 6). These results demonstrate that vlPAG Vglut2 neurons exert tonic control over thermal and mechanical nociceptive processing.

## Discussion

Here, we report that global chemogenetic activation of vlPAG neurons attenuates thermal nociception while inhibiting vlPAG neurons cause thermal and mechanical hypersensitivity. Using cell type-specific chemogenetic manipulations, we found that activating GABAergic, or inhibiting glutamatergic, neurons in vlPAG, causes thermal and mechanical hypersensitivity. In contrast, inhibiting GABAergic or activating glutamatergic neurons attenuates thermal sensitivity but has no effect on mechanical sensitivity. The differential effects on thermal and mechanical sensitivity suggest that distinct circuit elements within the vlPAG regulate these two sensory modalities. These results provide new insights into the functional role of GABAergic and glutamatergic neurons in the vlPAG in the modulation of nociception.

The vlPAG is known to be an essential component of neural pathways that mediate stimulation and stress-induced analgesia (Reynolds, 1969; Yeung et al., 1977; Jones and Gebhart, 1988; Morgan et al., 1989; Hohmann et al., 2005; Samineni et al., 2011). Consistent with these previous studies, we found that global chemogenetic activation of vlPAG neurons produced antinociceptive effects. We also show that chemogenetic inhibition of vlPAG neurons leads to nociceptive hypersensitivity, consistent with a bidirectional role of the vlPAG in the modulation of nociception (Moreau and Fields, 1986; Depaulis et al., 1987; Heinricher et al., 1987; Ness and Gebhart, 1987; Carstens et al., 1988; Jacquet, 1988; Jones and Gebhart, 1988; Jensen and Yaksh, 1989; Siegfried and de Souza, 1989; Behbehani et al., 1990; Carstens et al., 1990). The magnitude of antinociceptive effects observed after global chemogenetic activation of vlPAG neurons is modest relative to the robust effects produced by electrical stimulation of the PAG or by microinjection of morphine, GABA_A_ receptor antagonists, or glutamate agonists into the vlPAG (Reynolds, 1969; Liebeskind et al., 1973; Moreau and Fields, 1986; Depaulis et al., 1987; Ness and Gebhart, 1987; Carstens et al., 1988; Sandkühler et al., 1989; Siegfried and de Souza, 1989; Carstens et al., 1990). Such a difference could be expected if the number of neurons that are transduced with stimulatory DREADDs is small relative to the large number of neurons impacted by electrical or pharmacologic approaches.

Although the vlPAG has been extensively studied for its role in endogenous descending pain modulation (Reynolds, 1969; Liebeskind et al., 1973; Basbaum and Fields, 1978; Sandkühler et al., 1989; Behbehani et al., 1990; Vaughan et al., 1997; McGaraughty et al., 2003; Starowicz et al., 2007; Morgan et al., 2008; Waters and Lumb, 2008; Heinricher et al., 2009; Samineni et al., 2011; Wang et al., 2012; Ho et al., 2013; Lau and Vaughan, 2014; Tovote et al., 2016), previous studies have not yet determined how distinct subpopulations of vlPAG neurons modulate pain transmission. Inhibitory neurotransmission in the vlPAG is known to modulate nocifensive behaviors, since microinjecting a GABA agonist produces pronociceptive effects, while decreasing inhibitory neurotransmission by microinjection of GABA antagonists into the vlPAG produces antinociceptive effects to noxious stimuli (Moreau and Fields, 1986; Depaulis et al., 1987; Sandkühler et al., 1989; Budai and Fields, 1998; Morgan et al., 2003). Such studies led some to propose the vlPAG GABA disinhibition analgesia hypothesis (Basbaum and Fields, 1978; Fields, 2004). Corroborating this hypothesis, many studies have shown that mu opioid receptor agonists have direct inhibitory effects on GABAergic neurons of vlPAG. This causes analgesia when directly administered into the vlPAG, suggesting that inhibition of GABAergic vlPAG neuronal activity may be a major mechanism for opioid-induced analgesia (Chieng and Christie, 1996; Vaughan et al., 1997). The cellular mechanisms underlying the analgesic and hyperalgesic effects of manipulating inhibitory and excitatory neurotransmission in the vlPAG have not been directly evaluated (Moreau and Fields, 1986; Carstens et al., 1988; Jacquet, 1988; Sandkühler et al., 1989; Vaughan et al., 1997; Budai and Fields, 1998; Fields, 2004; Maione et al., 2006; Starowicz et al., 2007). It is not known how distinct neuron subpopulations in the vlPAG engage complex downstream circuits of the descending pain modulation pathway. For the first time, we show that chemogenetic activation of vlPAG GABAergic neurons causes hypersensitivity to nociceptive stimuli while their inhibition causes antinociception, consistent with the proposed role of GABAergic vlPAG neurons in pain modulation.

It has been hypothesized that GABAergic interneurons exert tonic inhibition of vlPAG glutamatergic neurons, which are thought to be output neurons that project to the RVM (Jacquet, 1988; Roychowdhury and Fields, 1996; Vaughan et al., 1997; Wang and Wessendorf, 2002; Morgan et al., 2008; Park et al., 2010; Hahm et al., 2011; Ho et al., 2013). These glutamatergic neurons have been hypothesized to play a role in an analgesic modulatory pathway (McGaraughty et al., 2003; Maione et al., 2006; Starowicz et al., 2007), but this has not been selectively demonstrated. In agreement with the GABA disinhibition hypothesis, our RNA-FISH studies show that GABAergic and glutamatergic neurons in the PAG are distinct populations. We also found that chemogenetic activation of vlPAG glutamatergic neurons is antinociceptive, whereas chemogenetic inhibition of these neurons is pronociceptive. Conversely, chemogenetic activation of GABAergic neurons in the vlPAG produces hypersensitivity while inhibition of these neurons produces analgesia.

Surprisingly, we find that activation of vlPAG glutamatergic neurons or inhibition of GABAergic neurons attenuated thermal but not mechanical sensitivity. While there are several possible explanations for this finding, we propose the hypothesis that distinct populations of inhibitory neurons regulate mechanical and thermal nociceptive modulatory pathways emanating from the vlPAG. Here, we posit the presence of tonically active GABAergic neurons regulating descending pathways for thermal nociception, while the population regulating mechanical nociceptive modulation might by quiescent. This would explain the difference in effects of activating GABAergic neurons or inhibiting glutamatergic neurons on mechanical nociception. It is possible that the differences in the effects of DREADD-dependent regulation are simply due to the basal state of the neurons in question. That is, if the neurons are quiescent at baseline, then activation of a G_i_-coupled DREADD might not affect descending modulation. Similarly, a neuron that is firing at a relatively high frequency at baseline might not be further stimulated by a G_q_-coupled DREADD. Future studies are necessary to determine the differences in neuronal populations and circuits that code for mechanical versus thermal sensitivity. It is also possible that these results are simply due to a ceiling effect in our von Frey measurements. That is, that von Frey testing is not able to detect analgesic effects at baseline. We do not favor this hypothesis, as treatment with analgesic drugs can indeed increase PWTs in mice (Anseloni and Gold, 2008).

We believe that our results should not be interpreted as absolute, and we recognize that the behavioral changes that we report should not be attributed to the entirety of either the Vgat or Vglut2 vlPAG neuronal populations. The PAG is comprised of molecularly diverse neuronal subpopulations that express fast neurotransmitters and/or neuropeptides (Mantyh, 1982; Moss and Basbaum, 1983; Moss et al., 1983; Smith et al., 1994). In the hypothalamus, recent genetic analysis of anatomically defined neurons has identified subpopulations that coexpress a variety of neuroactive substances that were previously thought to be exclusive to certain clusters (Romanov et al., 2017). Therefore, it is likely that vlPAG GABAergic and glutamatergic neurons can be further subdivided into subpopulations based on their genetic identity and physiology. Although we have only attempted to dissect the roles of glutamatergic versus GABAergic vlPAG neurons in pain modulation, future studies should examine the interplay between other neuronal populations within the vlPAG that can be defined with the expression of other makers, such as neuropeptides, to assess their roles in regulating nociceptive processing.

While the stimulation of vlPAG is predominantly associated with antinociceptive effects (Reynolds, 1969; Liebeskind et al., 1973; Carstens et al., 1988; Morgan et al., 1989; Sandkühler et al., 1989; Hohmann et al., 2005; Maione et al., 2006; Starowicz et al., 2007; Samineni et al., 2011), recent studies also identified facilitatory effects of the vlPAG in the maintenance of neuropathic pain (Pertovaara et al., 1996; Pertovaara et al., 1997; Heinricher et al., 2004; Guo et al., 2006; Lü et al., 2010). We show here that chemogenetic activation of GABAergic neurons or inhibition of glutamatergic neurons can lead to hypersensitivity to mechanical and thermal stimuli, suggesting that any disruption of the balance between activity of the vlPAG excitatory and inhibitory neurons might contribute to the maintenance of chronic pain (Hahm et al., 2011; Ho et al., 2013; Lau and Vaughan, 2014). Future studies should explore the plastic changes in GABAergic and glutamatergic neurons that might contribute to the maintenance of chronic pain.

The vlPAG has been shown to be instrumental in the descending modulation of pain processing. The vlPAG is known to form strong connections with the RVM, and the locus coeruleus (Beitz et al., 1983; Behbehani, 1995; Antal and Odeh, 1998; Odeh and Antal, 2001; Bowman et al., 2013). Based on the data we present here, it will be of great interest to determine which of these, or other, projection targets mediate the differential modulation of nociception by glutamatergic and GABAergic projections from the vlPAG.

## Synthesis

Reviewing Editor: Mary Heinricher, Oregon Health & Science University Decisions are customarily a result of the Reviewing Editor and the peer reviewers coming together and discussing their recommendations until a consensus is reached. When revisions are invited, a fact-based synthesis statement explaining their decision and outlining what is needed to prepare a revision will be listed below. The following reviewer(s) agreed to reveal their identity: Michael Morgan

The goal of the experiments reported in this manuscript was to provide an additional tests of a long-standing concept that glutamatergic neurons in the periaqueductal gray contribute to antinociceptive effects of activating this structure, while GABAergic neurons can exert a pro-nociceptive effect using a chemogenic approach. The findings are consistent with the large experimental literature supporting this idea, and are thus welcome. The concern remains that the manuscript is presented as if this is a new hypothesis, rather than confirmation of what was already generally well accepted. Although the Discussion has been revised to present a more scholarly analysis, the Abstract and Introduction were not.

A second significant conceptual issue is the assumption that GABAergic and glutamatergic neurons each comprise a functionally homogeneous neuronal “subtype.” This approach does not allow that type of conclusion. We can only say that there are at least some glutamatergic neurons that have a net antinociceptive effect when activated, and some GABAergic neurons that exert a net pro-nociceptive effect when stimulated.

It would also be worth acknowledging that the effects of the chemogenetic manipulations were modest in comparison with effects of opioids, electrical stimulation, or GABAA receptor antagonists. For example, line 349 states “we found that global chemogenetic activation of vlPAG neurons produced robust anti-nociceptive effects.” This is an overstatement. The antinociceptive effects are modest, with the largest change approximately 50% greater than baseline. The Discussion section should discuss the magnitude of this antinociception in the context of the much greater effects produced by microinjecting morphine, GABAA receptor antagonists, or glutamate agonists into the PAG. Robust antinociception is not evident here.

A significant methodological issue was not dealt with in the resubmission. Data are still analyzed using multiple paired t-tests as opposed to comparing different groups using ANOVA. It would be very simple to present the experiment as a chemogenic approach to test the hypothesis that glutamate neurons produce antinociception and GABA neurons enhance nociception. Experiment 1, which does not address this hypothesis, but instead apparently tests for effects of experimenter (at least according to the authors' response letter) could then be deleted. Subsequent data could be analyzed using a two-way ANOVA with repeated measures or even a one-way ANOVA comparing the effects of CNO across the three groups. Given that the baseline data were collected one week prior to the CNO test, this would not be unreasonable.

Some additional points

Abstract, line 62 says “...GABA activity causes nociception.” This is not what the data indicate. GABA activity facilitates nociception, but does not produce nociception independent of a noxious stimulus.

Abstract, line 63: The abstract ends by stating “Our findings offer insights into the distinct roles of excitatory and inhibitory neurons in vlPAG circuits involved in the descending modulation of pain, and raise the possibility that plastic changes in this circuit might contribute to development of chronic pain.” There are two problems with this sentence. First, this sentence provides no information. What are these insights? State them so the reader doesn't have to guess. Second, there are no data presented to support the conclusion that changes in this circuit might underlie the development of chronic pain. Bimodal modulation of pain occurs daily and does not imply a chronic pain state. This sentence should be removed.

Page 3, line 73 states “This is the first report to identify specific roles of these neuronal populations...” This is an overstatement. And, as already noted above the experimental approach used here cannot be used to conclude that these are functionally homogeneous populations.

Page 4, line 112: It is not obvious what is “unclear” about the role of GABAergic and glutamatergic neurons, especially given the citations that were added describing the role of these neurons. Given all the information that is presented, the reader doesn't know what is “unclear.” A much more satisfying approach would be to state that you will test the hypothesis that GABAergic neurons are “pro-nociceptive” and glutamatergic neurons are antinociceptive by selectively activating these neurons using “cell type-specific chemogenic manipulations.”

Line Minor errors:

Line 167: “All the electrophysiology experiments were performed the same age as in the behavioral studies.”

Line 223: “a nonparametric test or a Wilcoxon matched pairs test was used.” The Wilcoxon is a nonparametric test.

Figure 2: The scale bars on figures C and D go to 150%, but the maximum possible is 100%.

Figure 5: The scale bar for the paw withdrawal data (0 - 12 s) is not consistent with the scale used in Figures 1 and 4 (0 - 15 s).

Line 410 says “The vlPAG is known to form strong connections with the RVM, nucleus raphe magnus and the locus coeruleus.” The nucleus raphe magnus is part of the RVM. They are not separate structures.
